# Stress and the Brain: An Emerging Role for Selenium

**DOI:** 10.3389/fnins.2021.666601

**Published:** 2021-04-15

**Authors:** Daniel J. Torres, Naghum Alfulaij, Marla J. Berry

**Affiliations:** Pacific Biosciences Research Center, School of Ocean and Earth Science and Technology, University of Hawaii at Manoa, Honolulu, HI, United States

**Keywords:** selenium, stress, selenocompounds, glucocorticoids, selenoproteins

## Abstract

The stress response is an important tool in an organism’s ability to properly respond to adverse environmental conditions in order to survive. Intense acute or chronic elevation of glucocorticoids, a class of stress hormone, can have deleterious neurological effects, however, including memory impairments and emotional disturbances. In recent years, the protective role of the antioxidant micronutrient selenium against the negative impact of externally applied stress has begun to come to light. In this review, we will discuss the effects of stress on the brain, with a focus on glucocorticoid action in the hippocampus and cerebral cortex, and emerging evidence of an ability of selenium to normalize neurological function in the context of various stress and glucocorticoid exposure paradigms in rodent models.

## Introduction

**FIGURE 1:**
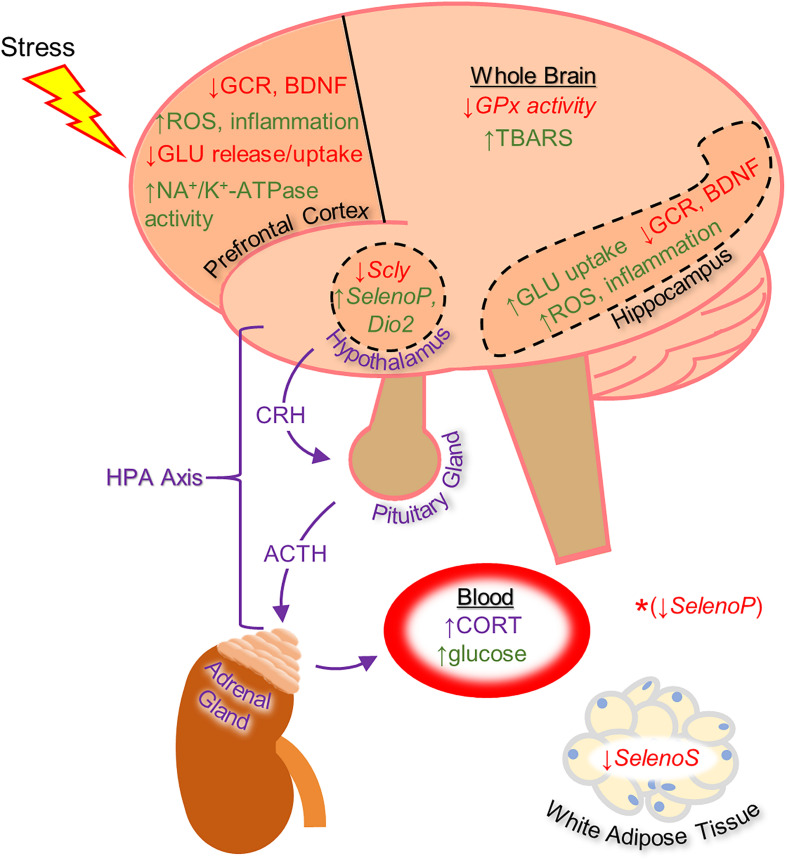
Diagram of the effects of heightened stress on the brain and other tissues as well as on the function of the hypothalamic-pituitary-adrenal (HPA) axis (in purple), based mainly on rodent studies. The effects shown include regulation of selenoprotein expression by glucocorticoid administration (shown with italicized text). *The synthetic glucocorticoid dexamethasone was shown to down-regulate Selenoprotein P gene expression in HEK-293 human embryonic kidney cells. With the exception of changes to selenoprotein expression, the effects listed on this diagram were shown to be reversible by selenium-based therapy. Up-regulation is indicated by green text and down-regulation by red text. ACTH, adrenocorticotropic hormone; BDNF, brain-derived neurotrophic factor; CORT, corticosterone; CRH, corticotropin-releasing hormone; Dio2, Iodothyronine deiodinase 2; GCR, glucocorticoid receptor; GLU, glutamate; GPx, glutathione peroxidase; ROS, reactive oxygen species; Scly, selenocysteine lyase; SelenoP, Selenoprotein P; SelenoS, Selenoprotein S; TBARS, thiobarbituric acid reactive substances.

The impact of stress on human health has been extensively investigated and the role of stress in disease pathology has become apparent over recent decades (Chrousos, 2009). The brain plays a key role in the response to stress, which includes higher order processing of stress-related information and an immediate physiological response executed by the hypothalamus, the proverbial “fight or flight response.” In addition to direct autonomic input to specific tissues, the stress response involves sending hormonal signals throughout the body via the hypothalamic-pituitary-adrenal (HPA) axis (depicted in Figure 1). Signaling along this pathway begins with the release of corticotropin-releasing hormone (CRH) by neurosecretory cells in the paraventricular nucleus of the hypothalamus. Upon stimulation by CRH, the anterior pituitary releases adrenocorticotropin-releasing hormone (ACTH), which then induces adrenal gland secretion of glucocorticoids into the bloodstream. Glucocorticoids comprise the main downstream component of the neuroendocrine response to stress and primarily serve to stimulate gluconeogenesis in the liver and lipolysis for energy production. They also suppress the inflammatory actions of the immune system and, thus, synthetic glucocorticoids are commonly prescribed in humans as anti-inflammatory medications. The autonomic component of the stress response, which includes vasoconstriction, inducing perspiration, and suppressing digestive activity, works in conjunction with glucocorticoids to provide an acute adaptation to stressful stimuli. Glucocorticoid receptors (GCR) are expressed in most tissues in mammals, however, and the physiological processes affected are wide-ranging. For example, both the hypothalamus and anterior pituitary express GCRs to provide negative feedback loops within the HPA axis by suppressing CRH and ACTH production (Godoy et al., 2018).

The brain is particularly sensitive to glucocorticoid levels and both acute and chronic stress (e.g., brief incidence of high stress or long-term exposure to low or moderate stress) can have deleterious effects on neurological function, including depressive symptoms and memory problems (McEwen, 2008; Lupien et al., 2018). In recent years, pre-clinical studies have demonstrated that the antioxidant micronutrient selenium has the capacity to alleviate the neurological repercussions of stress and exogenous glucocorticoid exposure. This review will provide an overview of the negative impact of stress on the brain, with a focus on glucocorticoid activity, and discuss the emerging evidence of the protective nature of selenium.

## Stress and the Brain

The reaction to stress, whether psychological or physical, can be defined as an attempt to regain homeostasis following a disruptive environmental stimulus (Chrousos, 2009). The short-term neuroendocrine response to stress provides adaptive benefits, but prolonged and repeated activation causes physiological “wear and tear” throughout the body, including the brain (McEwen, 2007). Excessive exposure of the brain to cortisol, which is the main active glucocorticoid in humans and can easily pass the blood–brain barrier, leads to deficits in learning and memory, attention, and emotional disturbances (Lupien et al., 2009). These neurological impairments are linked to dysfunction of the prefrontal cortex, the hippocampus, and the amygdala, brain structures that are integral to the processing of stress-related information and are particularly responsive to glucocorticoids (Lupien and Lepage, 2001). Following the discovery by McEwen et al. (1968) that corticosterone, the main active glucocorticoid in rodents, can act on the rat brain, it was noted that the hippocampus has the highest density of GCRs. Subsequently, the effects of glucocorticoids on the hippocampus and the relation to stress-induced cognitive dysfunction have been extensively characterized in animal and human studies throughout the years (Lupien and Lepage, 2001; McEwen et al., 2016; Lupien et al., 2018).

There are various ways that stress and glucocorticoid exposure can damage the brain. Early research in the field indicated that GCR over-activation causes neuronal damage by disrupting energy production, promoting energy over-consumption, and limiting glucose uptake into the cell (Sapolsky, 1986). Additionally, glucocorticoids can increase the risk of excitotoxicity by promoting the extracellular accumulation of glutamate in the hippocampus and prefrontal cortex (Stein-Behrens et al., 1994; Treccani et al., 2014). Oxidative stress is a prominent mediator of neuronal damage and dysfunction caused by psychological stress paradigms and exogenous glucocorticoid administration in rodents (Spiers et al., 2014). Glucocorticoids appear to make neurons more susceptible to oxidative insult by raising baseline levels of reactive oxygen species (ROS; McIntosh and Sapolsky, 1996; Behl et al., 1997).

## Selenium and Selenoproteins in Brain Health

The antioxidant trace element selenium is vital for overall human health and is especially important for brain function. Within the brain, selenium protects against oxidative stress, endoplasmic reticulum stress, and inflammation. There is also evidence that this micronutrient supports neurotransmission by maintaining redox balance (Solovyev, 2015). Selenium must be acquired through the diet and is most abundant in meats and legumes, as well as fruits and vegetables in trace amounts (Navarro-Alarcon and Cabrera-Vique, 2008). In the mammalian body, selenium is used to synthesize the amino acid selenocysteine (Sec), to be incorporated into selenoproteins, of which there are 25 types present in humans. Among the most well-characterized selenoproteins is the glutathione peroxidase (GPx) sub-family, responsible for reducing peroxide species, the thioredoxin reductases (TrxnR), and the iodothyronine deiodinases (Dio), which support thyroid hormone metabolism. In general, adequate selenoprotein expression largely depends on an organism’s intake of selenium, which is preferentially retained within the brain (Burk and Hill, 2009). Selenoprotein P (SelenoP), which is unique in that it has 10 Sec residues rather one, acts as a selenium carrier (Labunskyy et al., 2014). Following its secretion from the liver, SelenoP travels through the blood stream to be delivered to critical organs, such as the brain, where it interacts with apolipoprotein e receptor 2 (ApoER2) to deliver selenium (Burk et al., 2014). The brain is particularly dependent on selenium due to high rates of oxygen consumption and heightened susceptibility to oxidative stress (Steinbrenner and Sies, 2013). Insufficient selenium supply and lack of selenoprotein function have been linked to multiple brain disorders, including neurodegenerative diseases, which have been thoroughly discussed in previous reviews (Pillai et al., 2014; Solovyev, 2015; Varikasuvu et al., 2019; Zhang et al., 2019). Conversely, selenium has been suggested as a potential therapeutic agent in the treatment of Alzheimer’s disease (Solovyev et al., 2018), multiple sclerosis (de Toledo et al., 2020), and stroke (Alim et al., 2019).

Throughout the body, glucocorticoids have shown a capacity to alter antioxidant enzyme activity and expression (Dougall and Nick, 1991; Asayama et al., 1992; Kratschmar et al., 2012; An et al., 2016). In the brain, glucocorticoids can down-regulate several types of antioxidant enzymes, including GPx (McIntosh et al., 1998; Sahin and Gumuslu, 2004; You et al., 2009; Sato et al., 2010). Over the past several years, selenium has been shown to mitigate the negative impact of stress and glucocorticoid action in the brain.

## Selenium and Glucocorticoid Action in the Brain

A literature review was conducted with Web of Science and PubMed using the words “selenium” or “selenoprotein” combined with either “glucocorticoid” or “corticosterone,” as well as either with or without “brain”, yielding the following information. Early studies associating selenium and glucocorticoids focused on the physiological response to acute selenium challenge. Researchers discovered that injection of sodium selenite provokes a stress response, raising plasma corticosterone and glucose levels in rats within 30 min (Rasekh et al., 1991; Potmis et al., 1993). These studies established that acute elevations in selenium supply can activate the HPA axis. Conversely, dietary selenium deficiency blunts the ability of the adrenal gland to secrete corticosterone in response to ACTH administration (Chanoine et al., 2004). In addition to facilitating HPA axis activity, it was subsequently shown by Yilmaz et al. (2006) that selenium supplementation can reduce oxidative damage caused by the synthetic glucocorticoid prednisolone in the rat liver by maintaining reduced glutathione. More recent work by Beytut et al. (2018) found that pre-supplementation with sodium selenite prevented the rise in thiobarbituric acid reactive substances (TBARS) levels in rat brain caused by prednisolone injection. The authors hypothesized that glucocorticoids cause damage to neurons by inducing lipid peroxidation and that this occurs, at least in part, due to the ability of glucocorticoids to reduce antioxidant enzyme defense.

Work by Xu et al. (2020) suggests that dietary selenium may protect against stress-induced depressive symptoms. In this study, rats were subjected to social stress using a Chronic Unpredictable Mild Stress (CUMS) paradigm. While some developed depressive-like behavior and were classified as CUMS-sensitive, others did not and were, therefore, labeled CUMS-resilient. Analysis of trace element levels revealed that plasma selenium levels were lower in the CUMS-sensitive group, correlating low selenium levels with heightened susceptibility to stress-induced depressive-like symptoms. Additionally, an epidemiological study correlated low selenium intake with an increased susceptibility for developing major depressive disorder in humans (Pasco et al., 2012). It is important to note that these studies don’t show cause and effect, however. Still, the effects of selenium intake on the response to stress or glucocorticoid administration remains largely under-investigated.

Over the past several years, the protective role of selenium against the neurobehavioral consequences of glucocorticoids has started to come to light. In 2014, a report by Gai et al. (2014) described the ability of 3-(4-fluorophenylselenyl)-2,5-diphenylselenophene (F-DPS) to alleviate the anxiogenic- and depressive-like symptoms induced by chronic corticosterone administration in male Swiss mice. The organoselenium compound F-DPS is a selenophene, a class of selenium-containing aromatic compounds with antioxidant properties (Wilhelm et al., 2009; Tavadyan et al., 2017; Manikova et al., 2018), and was chosen for its antidepressant-like properties (Gay et al., 2010). One week of F-DPS treatment reversed the depressant- and anxiogenic-like behavior induced by 4 weeks of corticosterone administration. Glutamate uptake in the prefrontal cortex was reduced by corticosterone, which the authors noted was consistent with previous studies (Gourley et al., 2012) and likely contributed to the depressive-like phenotype. Administration of F-DPS during the final week of corticosterone administration restored glutamate uptake in the prefrontal cortex without causing any changes in vehicle-treated mice. These results parallel findings from clinical studies demonstrating the anti-depressive effects of the glutamatergic NMDA receptor antagonist ketamine (Yang et al., 2019). Additionally, F-DPS treatment was shown to reduce hippocampal serotonin uptake and monoamine oxidase A activity. Thus, promotion of serotonergic activity may have also contributed to the anti-depressive action of F-DPS (Gay et al., 2010; Gai et al., 2012). The authors concluded that these effects in the brain may have been mediated by an ability of F-DPS to normalize HPA axis function, as it was shown to reverse the rise serum corticosterone levels (Gai et al., 2014).

Following the work by Gai et al. (2012), another study explored the relationship between selenium and glucocorticoids in relation to memory. Zborowski et al. (2016) evaluated the potential of 4,4′-dichloro-diphenyl diselenide (*p*-ClPhSe)_2_, an organoselenium compound with antidepressant and memory enhancing properties (Gai et al., 2012) to alleviate the memory impairments caused by exogenous corticosterone. The researchers found that treatment with (*p*-ClPhSe)_2_ improved the performance of corticosterone-exposed mice in several memory tasks, while normalizing glutamate uptake in hippocampal slices. Intriguingly, there were no signs of toxicity caused by (*p*-ClPhSe)_2_, a common concern with selenium-based therapies, supporting the therapeutic potential of (*p*-ClPhSe)_2_. Later work revealed that (*p*-ClPhSe)_2_ is effective in reversing the depressive-like phenotype induced by chronic dexamethasone injections in mice (Heck et al., 2019). In this study, Heck at al. chose the synthetic glucocorticoid dexamethasone because it is widely prescribed as an anti-inflammatory in humans. In addition to preventing dexamethasone-induced depressive-like behavior and reducing ROS levels in the prefrontal cortex, (*p*-ClPhSe)_2_ normalized glutamatergic uptake in the prefrontal cortex, further implicating glutamatergic neurotransmission as a significant factor in the protective actions of selenium.

Several studies have also investigated the protective effects of selenium using an acute restraint stress (ARS) paradigm (Buynitsky and Mostofsky, 2009). This paradigm typically involves immobilizing subjects in a plexiglass restraint device with the goal of causing stress while minimizing pain. Previous research indicates that ARS works in part by targeting the antioxidant and inflammatory capacity of the brain (Sosnovskii et al., 1993; Spiers et al., 2016; Sayd et al., 2020). In a 2018 report, Sousa et al. described the ability of the selenocompound α-(phenylselanyl) acetophenone (PSAP) to counteract the effects of ARS (Sousa et al., 2018). Previous studies demonstrated that PSAP has GPx-like antioxidant activity (Cotgreave et al., 1992) and antidepressant-like capabilities in mice (Gerzson et al., 2012). Sousa and colleagues immobilized mice in a restraint device for 4 h, followed by a battery of behavioral tests 40 min later. A single dose of PSAP administered just after ARS and prior to behavioral testing reversed all of the behavioral changes induced by ARS, which included depressive-like and anxiogenic-like behavior, as well as an elevated sensitivity to pain. Administration of PSAP also decreased lipid peroxides and ROS in the hippocampus and cerebral cortex, which became elevated in response to ARS. Finally, PSAP prevented the rise in serum corticosterone caused by ARS, mimicking the results from previous studies indicating that selenium has a “normalizing” effect on HPA axis activity.

Several other selenocompounds have shown promising effects in stressed mice. Casaril et al. (2019) showed that 3-((4-chlorophenyl)selanyl)-1-methyl-1H-indole (CMI) can prevent ARS-induced depressive-like behavior in mice without affecting non-stressed subjects. Originally developed to combat atherosclerosis-associated inflammation by protecting extracellular matrix proteins from oxidative stress, CMI induces antinociceptive effects in mice by modulating serotonergic activity (Casaril et al., 2017b) and can reverse the depressive-like phenotype caused by lipopolysaccharide injection (Casaril et al., 2017a). Casaril identified multiple oxidative and inflammatory pathways that were activated by ARS and which CMI attenuated. The authors also revealed that CMI reversed the down-regulation of GCR expression in the prefrontal cortex and hippocampus caused by ARS that may have impaired the negative feedback loop of glucocorticoid secretion. Subsequent research by Pesarico et al. (2020) revealed that CMI also prevents the depressive-like phenotype caused by repeated forced swimming. The authors hypothesized that CMI acted by reducing lipid peroxidation in the prefrontal cortex and hippocampus. Domingues et al. (2019) obtained similar results while treating ARS-exposed mice with 3-[(4-methoxyphenyl) selanyl]-2-phenylimidazo[1,2-a] pyridine (MPI), a selenocompound with antioxidant and anti-inflammatory properties in the brain (Domingues et al., 2018). Administration of MPI attenuated the depressive- and anxiety-like phenotypes caused by ARS while preventing the induction of pro-inflammatory markers. Using a molecular docking simulation, the authors revealed that MPI may be capable of binding the GCR directly. Finally, Birmann et al. (2021) showed that yet another selenocompound, 3,5-dimethyl-1-phenyl-4-(phenylselanyl)-1*H*-pyrazole (SePy), protects against the anxiogenic-like and hyperalgesic effects of ARS. The authors reported that SePy, which has anti-depressive-like properties (Birmann et al., 2020), prevented the ARS-induced elevation of TBARS levels in the prefrontal cortex and hippocampus, while reducing plasma corticosterone levels. Additionally, SePy was predicted to bind the active site of GCRs, similar to MPI, using a computational model. The molecular effects of stress on the brain examined by these studies, as well as the impact on selenoprotein expression as discussed below, are summarized in Figure 1.

## Glucocorticoid Regulation of Selenoproteins

Glucocorticoids can regulate selenoprotein expression as reported by a handful of studies. For example, Rock and Moos identified a retinoid responsive element that can be regulated by dexamethasone to decrease *SelenoP* expression in HEK-293 cells (Rock and Moos, 2009). In another report by Kim and Kim (2013), dexamethasone was found to induce proteasomal degradation of Selenoprotein S (SelenoS) in 3T3-L1 murine preadipocytes, which the authors identified as necessary for adipogenesis. These studies highlight the diverse mechanisms through which glucocorticoids may differentially regulate selenoprotein expression in a tissue-specific manner.

Our knowledge of the ability of glucocorticoids to regulate the selenoproteins was recently expanded to the brain by Wray et al. (2019). In this study, chronic corticosterone administration increased gene expression of *SelenoP* and *Dio2*, while decreasing expression of the selenium recycling enzyme selenocysteine lyase (*Scly*), in the arcuate nucleus (Arc) of the hypothalamus, a brain region with high GCR expression. The authors focused on the metabolic effects of glucocorticoids, which include over-eating and excess weight gain (Vegiopoulos and Herzig, 2007; Perez et al., 2014). Interestingly, elevated serum SelenoP has been associated with diabetes and obesity (Misu et al., 2010) and Dio2 increases hypothalamic thyroid hormone availability (Bechtold and Loudon, 2007) to promote food intake (Coppola et al., 2007; Ishii et al., 2008; Varela et al., 2012). The finding that corticosterone down-regulated *Scly* draws an interesting parallel to whole-body Scly knockout mice, which exhibit an over-weight phenotype and heightened susceptibility to developing metabolic syndrome (Seale et al., 2012, 2015). Thus, long-term glucocorticoid action may promote positive energy balance, in part, by altering the expression of Scly and the selenoproteome in the Arc and other parts of the hypothalamus. In light of these findings, investigation of the interactions between glucocorticoids and selenium within the hypothalamus, and the relation to stress-related metabolic disruptions as well as downstream HPA axis function, remains a worthy course of investigation.

## Discussion

The majority of studies characterizing the protective role of selenium against stress and exogenous glucocorticoid administration have utilized various selenocompounds that were previously shown to have antioxidant activity. While the relative contributions of the selenium residues within each of these compounds to the overall therapeutic effect observed is not immediately clear, the protective results reported by the studies reviewed herein are striking (reviewed in Table 1). Developing synthetic compounds that incorporate selenium may, in fact, be a useful alternative to dietary selenium supplementation by providing the potential for tissue-specific targeting and limiting cytotoxicity. Still, dietary selenium remains an attractive potential treatment to counteract the oxidative effects of glucocorticoid action due its ease of delivery, and broad availability as an over-the-counter supplement. A comprehensive investigation of the role of selenium in the brain in response to stress, as well as the influence of glucocorticoid activity on the broader selenoproteome, however, is merited as this remains a major research gap. Additionally, investigating the apparent capability of seleno-therapy to normalize HPA axis function is instructive in order to understand the overall physiological implications. In conclusion, the interactions between glucocorticoids and selenium represent an emerging field with exciting potential for therapeutic development.

**TABLE 1:** Summary of the effects of selenium-containing compounds used in rodent models of stress.

Selenocompound/Species	Therapeutic Effects Against Stress in Rodent Studies		
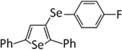 3-(4-Fluorophenylselenyl)-2,5-diphenylsel-enophene (F-DPS) (Gay et al., 2010)	(Gai et al., 2014) - Reversed depressant- and anxiety-like behaviors caused by CORT administration - Normalized serum ACTH and CORT levels - Lowered monoamine oxidase-A activity in the PFC - Augmented synaptosomal serotonin and restored GLU uptake in PFC		
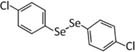 4,4′-dichloro-diphenyl diselenide (*p*-ClPhSe)_2_ (Gai et al., 2012)	(Zborowski et al., 2016) - Restored spatial and non-spatial memory dysfunction caused by CORT administration - Reversed GLU uptake augmentation in HPC slices	(Heck et al., 2019) - Prevented depressive-like behavior induced by dexamethasone administration - Reduced ROS; Restored CAT, SOD activity. - Restored GLU uptake and release; reversed elevation of NA^+^/K^+^-ATPase activity in PFC.	
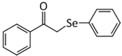 α-(phenylselanyl) acetophenone (PSAP) (Gerzson et al., 2012)	- (Sousa et al., 2018) Prevented depressive- and anxiety-like behavior caused by ARS - Prevented the associated elevation in pain sensitivity and allodynia (perceiving normally non-painful stimuli as painful) - Normalized serum CORT levels - Reduced ROS, lipid peroxidation, nitrite, and nitrate levels in the CC, HPC		
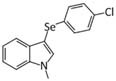 3-((4-chlorophenyl)selanyl)-1-methyl-1H-indole (CMI) (Vieira et al., 2015; Casaril et al., 2017b)	- (Casaril et al., 2019) - Prevented depressive-like behavior caused by ARS - Normalized serum CORT levels - Reduced ROS, lipid peroxidation, and nitric oxides in the PFC, HPC - Restored CAT activity in the HPC - Prevented down-regulation of GCR and BDNF, and up-regulation of inflammation in the PFC and HPC		- (Pesarico et al., 2020) - Prevented depressive-like behavior caused by the repeated forced swimming test - Normalized serum CORT levels - Prevented TBARS elevation in HPC - Restored SOD activity in HPC - Reversed the up-regulation of CAT activity in the PFC and down-regulation in the HPC
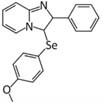 3-[(4-methoxyphenyl) selanyl]-2-phenylimidazo[1,2-a] pyridine (MPI) (Domingues et al., 2018)	- (Domingues et al., 2019) - Prevented anxiogenic-like behavior caused by ARS - Normalized plasma CORT levels - Prevented the rise in plasma glucose levels - Prevented elevation of TBARS, ROS, and nitrate/nitrites in the PFC and HPC - Prevented elevation of inflammatory markers in the PFC and HPC - Prevented the down-regulation of BDNF in the PFC and HPC - May be capable of binding GCR directly		
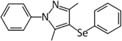 3,5-dimethyl-1-phenyl-4-(phenylselanyl)-1H-pyrazole (SePy) (Birmann et al., 2020)	- (Birmann et al., 2021) - Attenuated anxiety-like behavior, allodynia, and hyperalgesia caused by ARS - Normalized plasma CORT levels - Reversed the elevation of ROS and TBARS in the PFC and HPC - Restored SOD activity in the PFC and HPC - May be capable of binding GCR directly		
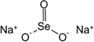 Sodium Selenite (Na_2_SeO_3_)	- (Beytut et al., 2018) - Reduced total brain TBARS induced by prednisolone administration - Restored brain GPx activity and levels of reduced GSH - Did not, however, prevent the reduction in CAT activity		

*(**Left column**) Selenium-containing compounds used in the reviewed studies and references for preceding studies with those compounds. (**Right column**) Ameliorative effects of treatment with the selenocompounds against the neurological and physiological impact of various stress paradigms. ACTH, adrenocorticotropic hormone; ARS, acute restraint stress; BDNF, brain-derived neurotrophic factor; CAT, catalase; CC, cerebral cortex; CORT, corticosterone; GCR, glucocorticoid receptor; GLU, glutamate; GPx, glutathione peroxidase; GSH, glutathione; HPC, hippocampus; PFC, prefrontal cortex; ROS, reactive oxygen species; SOD, superoxide dismutase; TBARS, thiobarbituric acid reactive substances.*

## Author Contributions

DT and NA wrote the manuscript. All authors revised the manuscript.

## Conflict of Interest

The authors declare that the research was conducted in the absence of any commercial or financial relationships that could be construed as a potential conflict of interest.
